# Ten simple rules for productive lab meetings

**DOI:** 10.1371/journal.pcbi.1008953

**Published:** 2021-05-27

**Authors:** Nigel Golden, Kadambari Devarajan, Cathleen Balantic, Joseph Drake, Michael T. Hallworth, Toni Lyn Morelli

**Affiliations:** 1 Department of Environmental Conservation, University of Massachusetts, Amherst, Massachusetts, United States of America; 2 Northeast Climate Adaptation Science Center, Amherst, Massachusetts, United States of America; 3 Organismic and Evolutionary Biology Graduate Program, University of Massachusetts, Amherst, Massachusetts, United States of America; 4 U.S. Geological Survey, Amherst, Massachusetts, United States of America; Carnegie Mellon University, UNITED STATES

## Introduction

Lab meetings, recurrently scheduled interactions among research group members, and collaborators with diverse objectives, are integral to academia and scientific research. Establishing and cultivating a lab culture through regular meetings can have a profound impact on the productivity of a group as well as of individual lab members. Here, our framework for productive lab meetings is participant-centered; productive lab meetings are spaces that are healthy, safe, and allow for individual participants to have their objectives met. These objectives are identified and defined by the participants with feedback from their peers and principal investigator. In other words, productive lab meetings help participants to best achieve their research and academic goals considering their personal context (e.g., career stage) and identities. Productive lab meetings provide a safe environment for group members to practice communication skills, decision-making, problem solving, critical thinking, and collaboration [1]. They can influence research and discourse, within and beyond the lab. Productive lab meetings foster a sense of participation, integration, and inclusion among a diverse lab community, and leverage the diversity of experiences and skills of lab members. Thus, the group’s productivity and success increase, helping to increase diversity in science [2,3], boost scientific creativity, and facilitate problem solving [4].

**Fig 1:**
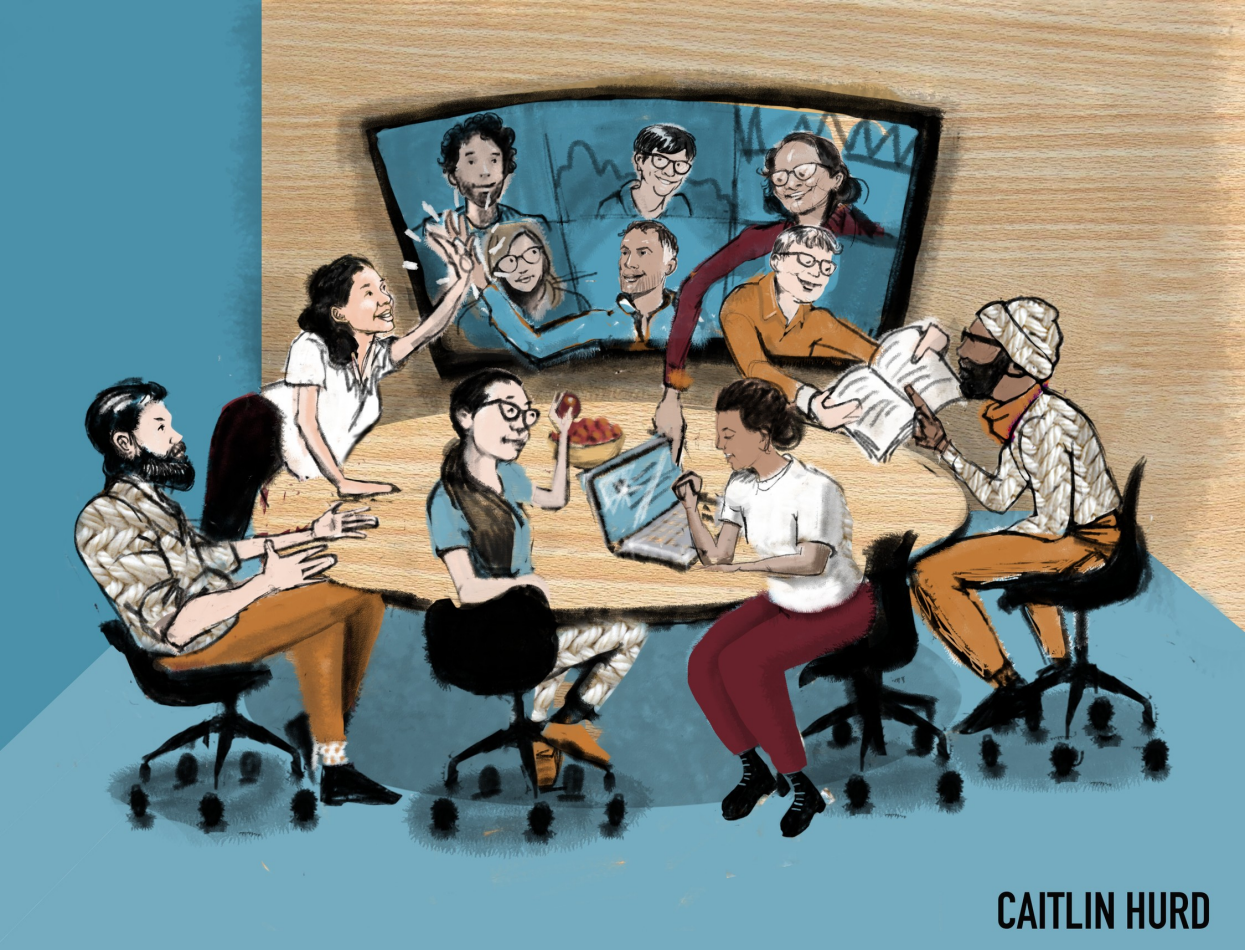
At the time of this publication, our lab meetings typically consisted of one principal investigator, 5–10 graduate students, one postdoctoral fellow, some undergraduates, and a few visiting students. Prior to the COVID-19 pandemic, the meetings had a mix of in-person and virtual attendees to maximize inclusion and accessibility, and attendees frequently brought snacks to share if they desired to do so. At the onset of the pandemic, our lab meetings demonstrated adaptability (see Rules 3 and 10) by shifting into a fully virtual environment (Box 1). In weekly meetings throughout the semester, our typical format has lab members rotate as meeting facilitators each week by leading a journal article discussion, discussing a topic of interest to lab members (e.g., career guidance, science communication, issues of diversity, equity, inclusion, and justice), or getting feedback on a current research effort (e.g., manuscript draft, upcoming presentation, defense rehearsal). Each meeting begins with an icebreaker to foster supportiveness and a brief reflection on our discussion ground rules to collectively recommit to meetings that are open, mindful, and respectful. *Illustration credit*: *Caitlin Hurd*.

The aim of this article is to delineate 10 simple rules on how to achieve productive lab meetings. We use the term “meeting” interchangeably to represent both the single meeting event and the overarching concept of the recurring meeting. In this article we speak from our experience, as a lab group at the University of Massachusetts that meets regularly (Fig 1). Although the rules are mostly tailored toward academic or research institution settings, insights can be gained for other contexts. We believe these rules are applicable across a diverse set of labs and lab structures. For example, while many members of our current lab have remained constant for many years, the lab composition has changed as various undergraduate students, graduate students, postdoctoral fellow, visiting professors, and other faculty have joined and/or moved on. Throughout these experiences, lab rules, presented in modified form here, proved flexible and adaptable enough to be useful in helping guide productive lab meetings. Note that this article is written for principal investigator/s (PI), students, postdocs, and other lab members; it takes the whole lab group to succeed. The key to planning productive lab meetings boils down to discussing and determining as a team the answers to why, who, what, where, when, and how: Why are lab meetings important for the functioning of the lab? Who will participate? What will be the focus of lab meetings? When and where should the lab meetings occur? How should each meeting be structured and carried out so that the goals and objectives of the lab and its participants are met?

**Box 1. How to have productive virtual meetings**

Virtual lab meetings have become critical to academic life due to the COVID-19 pandemic. As entire nations entered quarantine and enacted shelter-in-place rules, many transitioned abruptly to engaging in virtual meetings. These workers quickly started experiencing what has been described as “Zoom fatigue” [20]. Among other issues, virtual meeting platforms may inherently exacerbate problems with reading emotional cues and increase feelings of frustration due to delays in digital signals between participants [21]. Virtual meetings are inherently limited because much of the body language key to the interpretation of messages is often lost in digital presentations/settings [22] which could result in emotional stress.

The transition to online meetings has unveiled a complex set of inequities that impact how we work. Much of the ability to work remotely depends on access to a robust and reliable telecommunications network. A “digital divide” separates those with access to high-speed internet from those in rural communities or lower-income households who don’t have it [23]. This divide highlights inequities, particularly for lab members who do not have the infrastructure to work effectively from home. Moreover, with school and many group childcare options canceled, those without access to family networks and expensive individualized childcare are struggling [24]. Moreover, financial inequities can be on full display when we virtually invite people into our homes. The suite of financial, physical, and psychological burdens that virtual meetings can present places an emphasis on objectives, roles, inclusion, biases, and overall adaptability (see Rule 10), as laid out in this manuscript.

Nevertheless, virtual or hybrid lab meetings will likely be with us for the foreseeable future [25], and they offer several advantages. Since most academic responsibilities are tied to teaching and learning, lab meetings can be a place to practice thoughtful approaches to effective instructional design that increases engagement in online settings (e.g., breakout rooms, live survey/polls, think-pair-share). Moreover, more studies are incorporating researchers across institutions and international boundaries to support inclusive scientific communities [26]. Furthermore, participants from online-based conferences reported that the experience was more inclusive, diverse, and egalitarian [27]; they also have smaller carbon footprints than in-person conferences [27]. Additionally, immigration barriers have become more common across the globe in response to political and global health concerns; virtual meetings allow those unduly affected to maintain important social and academic connections in a trying time (although time zone differences need to be accounted for).

As with in-person meetings, it is important to establish rules and roles in the videoconferencing environment. If the group is small, microphones may remain unmuted, but one advantage to the videoconference environment is that an unmuted microphone can act as a subtle signal for someone wanting to speak. As with all meetings, good facilitation is key. Flexibility and adaptability are also important; breeding a culture of keeping videos on is important but allowing for exceptions for those who are having an off week or who do not have adequate bandwidth is also beneficial. The chat box can also help more introverted members to contribute their thoughts or provide more space for conversation given time constraints.

We divide our 10 simple rules into 3 categories: (1) process rules; (2) group responsibility rules; and (3) individual responsibility rules. Process rules (Rules 1, 2, and 3) outline a strategy to provide the structural foundation for productive lab meetings. Group responsibility rules (Rules 4, 5, and 6) review ways that participants can collectively cultivate a productive lab meeting environment. Individual responsibility rules (Rules 7, 8, and 9) outline ways each lab member can modulate their own participation to foster lab meeting productivity. Rule 10 is applicable across all 3 rule categories.

### Rule 1: Define lab mission and objectives

**Fig 2:**
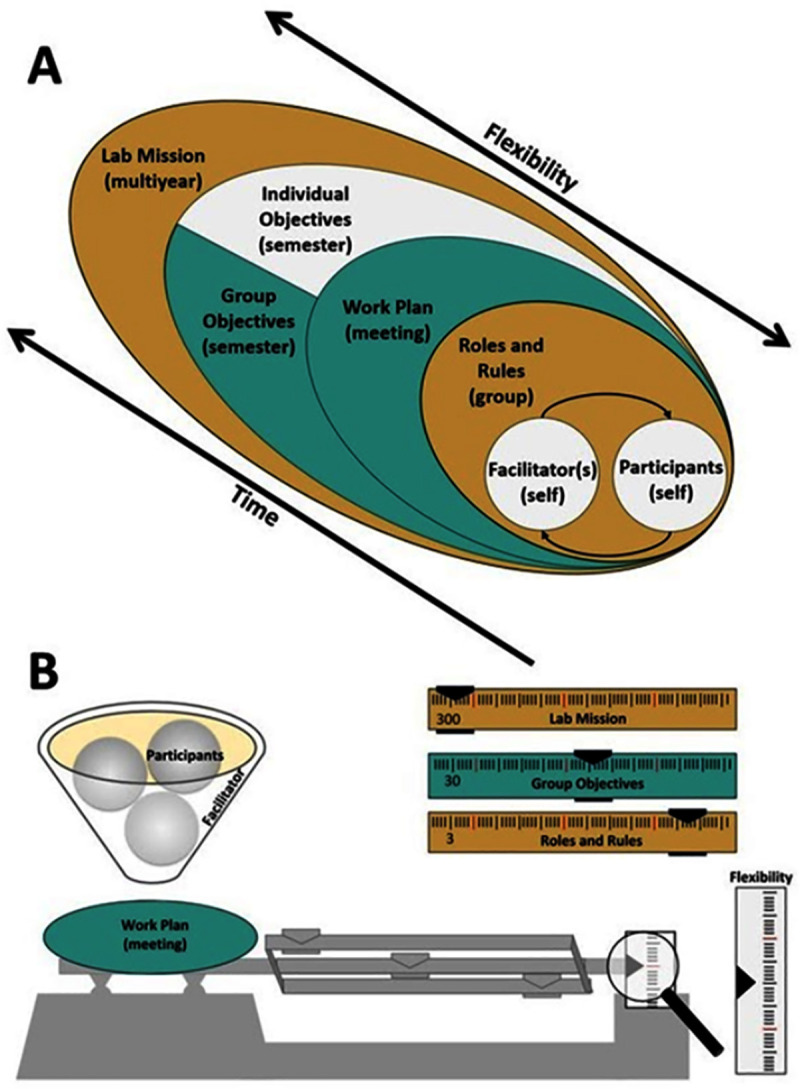
There is no “one size fits all” approach to design or implement productive lab meetings. Here are 2 different examples of how to envision lab meetings, the structures of goals, timelines, and collective or individual responsibilities. (A) A hierarchical approach to the design and implementation of productive lab meetings. This version emphasizes a nested design to represent how each component (or each Rule) influences and informs each other. These can be roughly grouped together based on how they fit into categories of process rules (mustard), group responsibility rules (turquoise), and individual responsibility rules (white), respectively. Although meeting dynamics may play out at several temporal and structural scales, one essential aspect of meeting culture weaves through all layers: being flexible and adaptable (See Rule 10). (B) An integrated approach to visualizing productive lab meetings wherein all components balance. This “balanced” approach illustrates how each aspect of lab meetings and the way in which they are conducted (such as through a set of Ten Simple Rules) all work together to get the desired end result. The facilitator moderates the participants as part of the meeting (work plan/lab session). The lab mission carries the most weight (heavier measurement), the group objectives carry less weight but are important, the roles and rules involve frequent, small, finely tuned changes to get to the end goal/s. The target (under the magnifying glass) can change through time indicating flexibility and adaptability.

As with any meeting we encounter in academic settings (e.g., committee meetings, faculty meetings, advisor meetings), lab meetings will be most effective and productive if they have clearly articulated objectives and fit within the overall lab mission (Fig 2) [5]. Many research enterprises and organizations have a “mission” statement. For instance, the mission of the United States Geological Survey (USGS) is “to monitor, analyze, and predict current and evolving dynamics of complex human and natural Earth-system interactions and to deliver actionable intelligence at scales and timeframes relevant to decision makers” (https://www.usgs.gov/about/about-us/who-we-are). Considering the expectations for a specific meeting within the context of the lab’s mission will help lab groups articulate goals and expectations.

Participants’ objectives can then inform why you have lab meetings and how to conduct them, and thus make them most productive for lab members [6]. Brainstorming and setting objectives can help unearth insights about how to design and run the meetings to the maximum benefit of your group [7]. A lab meeting’s underlying purpose will be best achieved if framed using a people-centric approach where the objectives are driven by the participants [8]. The expectations of productivity may differ between individual meetings, or among individuals within a specific meeting. Thus, objectives must be clearly defined and be measured on an individual basis as well as from the perspective of the entire lab group. In our experience, having clearly defined objectives early on and revisiting them as necessary creates a foundation from which to select the appropriate activities and content to meet those objectives. For instance, the broad objectives of a lab group could be to enhance scholarly learning and practice critical thinking within an inclusive venue.

Lab meetings that have no direct link to the lab’s mission or lab members’ objectives can miss important teaching/learning opportunities, be poorly received, and cause a loss of momentum for participants. It is essential that the lab’s PI and members carefully work together to ensure the needs of participants are met and accomplish the stated objectives. A common lab meeting objective may be to advance the scholarly work of lab members. However, discussions need not be limited to research-related topics. Other objectives could be to cultivate a sense of group identity and belonging and to establish a positive community that influences how lab members interact outside of lab meetings [9–11]. We suggest that lab meeting objectives be shaped according to the career stage of the participants, type of institution, and discipline of the lab; thus, it is likely to change with time.

### Rule 2: Identify roles and rules

For a lab meeting to run efficiently, all participants should have a clear understanding of their individual roles (Fig 2), as well as the rules of the meeting environment. Long-term meeting roles and ground rules might be developed and agreed upon by the meeting group based on the lab mission and group objectives. For example, our lab group crafted a set of meeting guidelines that helped identify roles and rules for our group meetings, occasionally revisiting and updating them to reflect shifting needs over time. A few basic foundational rules we address include defining when, where, and how frequently meetings occur, and whether all group members are expected to attend each meeting. Important role definitions include clarifying expectations around who in the meeting group provides input to decisions and meeting planning, and who has final decision-making power (which may be the PI, or may be collective depending on the group’s structure). A group that crafts a set of lab guidelines together may consider additional questions: How will our roles/rules norms ensure that meetings are inclusive and accessible to all attendees (see Rule 3)? For example, can members attend remotely if needed (see **Box 1**)? Will meetings have a set structure with a recurring agenda? Who is responsible for ensuring that meetings begin and end on time? Are snacks expected at meetings and, if so, are they paid for out of a lab budget or by individual members? Who is welcome to attend the meetings—are these strictly for students and postdocs advised or supervised by the lab PI, or can other groups join to foster additional collaboration and community?

Framed within the larger lab mission, the roles and rules of a single meeting will vary based on that semester’s individual and group objectives and that particular meeting’s objectives and work plan as delineated in Fig 2. To illustrate, lab members may trade off acting as the “meeting facilitator,” who has a responsibility to the rest of the lab (the “participants”) to ensure a productive meeting by developing a work plan and providing a structure through which objectives are communicated. For example, if the meeting facilitator is giving a practice talk in preparation for an upcoming presentation, they can let the group know where they are looking for input, i.e., clarity of message, delivery, flow, etc. If discussing a facilitator’s manuscript draft, the facilitator might give pointed advice on what they would like feedback on, actively listening so that participants feel that time providing feedback was well spent. Depending on the meeting objectives, the meeting facilitator can arrange how activities are organized (e.g., slides, whiteboard use, work in small groups or pairs). In addition to being actively engaged in the meeting, participants who are not meeting facilitators may have their own designated roles (e.g., timekeeper, notetaker, or simply a supportive group member prepared to engage thoughtfully with the content).

If the role of the meeting facilitator comes with responsibility, so does the role of meeting participants (see Rules 3 to 8). Just as the meeting facilitator should come prepared to provide a presentation or facilitate a discussion or activity, so should the rest of the lab come prepared to actively participate. Coming to lab meetings prepared (for example, reading facilitator-provided material) is a social contract, which shows respect and enables participants to meet their objectives. Both meeting facilitators and participants should have a reasonable expectation to provide or respond to post-meeting resources, comments, or questions in a timely fashion. In this way, pre-meeting preparation, active meeting participation, and post-meeting follow-up make the most of the precious time that members dedicate to each other.

### Rule 3: Be accessible and inclusive

Accessibility and inclusion go hand in hand. Accessibility here refers to the degree to which lab meetings are attended by and welcoming/supportive to as many people as relevant. There are different dimensions of diversity and accessibility to consider when running lab meetings. In some contexts, these terms refer to differently abled, neurodiverse, or special-needs learners [12,13]; in others, they are used in the context of racial, gender, cultural, and socioeconomic diversity [14,15]. Disabilities and personal circumstances that limit participation in lab meetings are diverse; they can be visible or invisible [16]. While graduate school is stressful for all, students from underrepresented groups are confronted with the additional challenges of marginalization within the divisive and competitive environment of academia despite being more innovative than majority students as indicated by the diversity–innovation paradox [17]. An intentional focus towards accessibility and inclusion reveals how facets of our personal identity shape our opportunities to succeed and be productive.

Working to be accessible/inclusive is necessary and rewarding, and though resources abound for navigating accessibility challenges (e.g., ITACC Project Advisory Committee 2012) [18], there is no simple list of rules to follow. Here, we provide some guidance, stemming from our own experiences, for where PIs and lab meeting participants can start:

1. Set up lab meetings so that participants are able to manage their own access needs. For example, for health or childcare needs, provide access to lab meetings at a secondary location or online (see Box 1).
2. Embed assistive technology to support engagement in lab meetings [19]. Closed captioning is readily available in several desktop and online presentation tools. Simple steps include using easy-to-read fonts and font sizes, using sufficient color contrast, and color blindness-friendly palettes.
3. Use inclusive language, appropriate pronouns, and people’s preferred terms. Inclusive language can be as simple as using substitutes such as “folks” or “everyone” instead of “guys.”

Access and inclusion can be increased by taking a proactive approach. The key is to be flexible and adaptable (see Rule 10), make accessibility a priority, and enable participants to feel comfortable getting what they need. To help cultivate an inclusive and accessible environment in lab meetings, participants and especially PIs should keep abreast of the popular discourses around accessibility, inclusion, and implicit biases.

### Rule 4: Be supportive

Feelings of isolation and varying degrees of depression, anxiety, and disengagement are commonly documented among students, especially for students from underrepresented groups who often lack access to professional networks [28–33]. In the absence of a supportive community, students may disengage and prematurely exit the program [34], as well as suffer from stress-induced health effects. Thus, it is imperative that lab meetings are structured in a way that all students are supported and are supportive of each other, both in the professional and personal dimensions of their experience in academia [35,36].

Types of support can be emotional (such as trust, empathy, and encouragement) and/or professional (such as helping and mentoring according to specific needs, advice-giving, and providing feedback) [37]. Ideally, the lab meeting is intentionally designed from a people-centric approach where members want to participate because they feel a sense of community, camaraderie, and support. Lab meetings should be a place that is safe physically, intellectually, emotionally, and culturally, where members can celebrate their accomplishments and share disappointments, both big and small, with the group. An environment that encourages collaboration (and discourages toxic competition) is critical, albeit difficult to foster [1]. One small way our lab group promotes supportiveness is through sharing our successes and accomplishments (personal or professional) at the beginning of each weekly meeting. This acts as our meeting icebreaker and gives lab members the opportunity to share recent achievements both large and small, which might be professional (such as submitting a manuscript or getting an award) or personal (such as celebrating progress in a hobby or recommending a good recipe).

### Rule 5: Be respectful and practice civility

In interacting with each other during lab meetings, strive for creating respectful and humanistic spaces. In other words, practice civility and be respectful to everyone in the lab group. Research on workplace incivility makes it clear that an intentional focus on civility and respect is beneficial as it improves interpersonal relationships between participants (see Rule 4; [38]) and productivity [39,40], whereas workplace incivility can result in heightened levels of burnout and withdrawal [41]. This is especially true when the root of that incivility is based on intersecting marginalized identity markers in academia [42,43] (see Rule 9).

Here, we consider the lab meeting setting to be part and parcel of academic workplaces. There are standards, protocols, or rules with regards to how we treat each other in most other aspects of our professional lives, including conferences, fieldwork, workshops, laboratories, and classrooms. Lab meetings deserve this consideration as well. Distracting behaviors—being curt, disrespectful, insensitive, or snappy—can strain group dynamics and potentially lead to less productive discussions. Incivility includes interrupting others, tuning out and being distracted, and having side conversations. These behaviors and microaggressions can have compounding harmful effects over time. To reinforce workplace civility, our lab meetings begin by reflecting on a rule from our shared meeting guidelines document, modifying as necessary each semester with changes in lab composition (see Rule 10). Thus, the “rules” are regularly engaged with and kept relevant.

### Rule 6: Manage conflict

It is important to foster an environment of collegiality, collaboration, and support within a lab group, but creating a cohesive team of lab group members can be challenging. These challenges may extend to lab meetings, which bear witness to the unique internal ecosystem of energy levels, stressors, opportunities, constraints, strengths, and weaknesses each individual member brings to a meeting. Moreover, most lab groups include a diversity of stages and priorities. Conflict may arise during a lab meeting despite our best intentions, even when objectives and rules have been clearly defined, and even when participants are making good faith efforts to be open-minded, supportive, present, respectful, and conscious of their biases [44].

Be careful to distinguish between dissent and conflict. A disagreement by itself is not a conflict, and neither dissent nor conflict should be managed in a way that forces individuals to conformity [45]. Dissent can be a hallmark of a thriving and stimulating lab meeting environment where diverse perspectives are valued [46] and is especially important to research. Signs of escalation into conflict include silence, palpable tension, adversarial behavior, and aggressiveness [47]. Such escalation might originate from personality conflicts, resource conflicts over shared lab equipment, or disagreements over responsibilities, authorship, or intellectual ownership; for example, a lab member may feel another member has stolen an idea after reading a draft manuscript for a lab meeting [48]. The PI can guide meeting conflicts toward resolution by summarizing and clarifying dissenting positions, encouraging listening, and making sure lab members understand one another’s perspectives [49]. In fact, by fostering a sense of collaboration rather than competition (for example, by following Rule 4: Be supportive), regular lab meetings can intrinsically reduce lab conflict by increasing communication opportunities [1,48,50]. Potential authorship conflicts can be minimized by developing and maintaining authorship guidelines for the reference of lab members. Thus, to prepare collectively for conflict mitigation, the forward-thinking lab group might develop meeting guidelines together (perhaps inspired by these 10 simple rules!) to serve as a community touchstone when conflicts do arise during meetings [46].

### Rule 7: Be open and curious

Many of the guidelines we have developed for our own meetings speak to the spirit of being open and curious. Though open-mindedness feels effortless when the idea at hand is consistent with our existing views (see Rule 9: Be aware of biases), a greater challenge is consciously remaining receptive to suggestions, concepts, and critiques that contradict one’s individual views and lived experiences to revise one’s viewpoint (i.e., learn) [51]. A commitment to open-mindedness during meetings can foster curiosity (active information-seeking) and lead to new ideas and knowledge creation, in addition to helping preempt conflict (see Rule 8) [52], and thus improve lab meeting productivity.

Openness and curiosity can extend to the lab meeting content itself; a willingness to explore outside the lab discipline can foster a more productive environment and inclusive community culture. For instance, dedicating time to discussing topics outside of the strict lab discipline—such as research ethics, sexual harassment in STEM, and professional development—can help foster a more robust, creative, and healthy lab community both within and beyond the meeting environment [1,14,53].

### Rule 8: Be mindful and present

Lab meetings are more productive when group members are present, engaged, and mindful, whether in person or on video. This includes being an active listener, moderating the frequency and extent of one’s own participation during meetings to allow space for others, while actively contributing to the discussion. Eliminating distractions, such as closing unrelated laptop/browser windows or leaving the phone silent/off when possible, can enable members to be fully present during lab meetings and to more effectively engage in (or lead) the conversation.

In order to critique ideas and not people, we encourage phrases like “I disagree with your argument” or “Have you considered…?” rather than “You’re wrong,” when providing feedback. Additionally, when receiving critiques, start with the assumption that people mean well. We need to be mindful of our initial responses (which may be defensive), take a breath, and try to remember that critiques are not personal. Furthermore, being mindful includes being cognizant of one’s own body language and other nonverbal behavioral cues during lab meetings; participants can engage in positive, open, and inviting body postures to promote broader participation and comfort during meetings. For instance, perhaps a meeting participant has something to contribute but hasn’t found the appropriate opportunity to do so. Being aware of any participant cues and having mechanisms to ensure broader participation (such as “raising hand” or using the chat window in virtual meeting environments to order participants) can help promote a more equitable and mindful discussion. Furthermore, nonverbal cues during meetings are important and help build member confidence. Shaking their head in agreement or nodding to acknowledge understanding are signs a member is involved and actively listening to the presenter. These types of behavioral cues are equally important during virtual meetings as well but may need to be accentuated or exaggerated to convey the same level of effectiveness; emojis and digital responses can help.

### Rule 9: Be aware of biases

In academic settings (including lab meetings), there are identity-based biases that create hostile environments, impact individual productivity, and thus hinder success [54]. These biases are informed both by an individual’s social environment (e.g., political contexts, social norms and values) and their personality traits. This includes perceptions of aspects of identity that include disabilities, gender, race, ethnicity, sexual orientation, and socioeconomic status. Extensive research shows that these perceptions affect our interactions and how we receive information [55]; for example, faculty (regardless of gender identity) are often biased against female-identified students [56].

Such biases can result in forms of "subtle discrimination," or microaggressions, and incivility (see Rule 5) that can be just as damaging to the recipient as overt forms of discrimination [57], highlighting the need for bias training to reduce harm, as well as the need for interventions to address microaggressions when they occur [58]. It is therefore not enough to simply be aware of biases; it is important to confront our biases on how we take in information, including perceiving others, and then commit to actively unlearn these biases, in order to create a more diverse and inclusive, and thus more productive lab meeting setting [59]. The first step can be for lab members to intentionally discuss implicit biases in a lab meeting (see Rule 3); for example, lab members can facilitate an open dialogue on combating biases within and outside of lab meeting interactions. Other strategies on the recognition of biases can be accomplished through education, facilitated discussions by professionals, and/or implicit biases tests (e.g., https://implicit.harvard.edu/implicit/takeatest.html). Ultimately, the goal, which can be abetted by existing educational and training programs [60,61], should be to reduce these biases.

### Rule 10: Be flexible and adaptable

Although we have set out to highlight a set of best/better practices, we understand that life can be unpredictable (see Box 1), which leads us to our last rule. As we have noted, the design and implementation of lab meetings should allow for flexibility and adaptability. As participants rotate in and out of lab meetings and as their needs change, it will be useful, and indeed beneficial for the culture of the lab group, to occasionally or even systematically (e.g., at the start of each academic year) assess what works and what could be improved. Thus, roles and rules might change as members achieve different stages, or biases might be revealed as new members join. For instance, many of us have been impacted to various degrees by the COVID-19 pandemic. Working remotely has now become integral to our professional lives. This has resulted in unusual work scenarios that required modifying and adjusting our lab meetings, including changing the meeting time to accommodate multiple time zones and holding meetings throughout the summer to increase intellectual and emotional support during these challenging times. The COVID-19 pandemic has highlighted how important it is to be flexible and adaptive, for the physical, emotional, and professional well-being of the lab community.

## Conclusions

Lab meetings can be some of the most memorable and rewarding moments in our academic journeys. When navigated thoughtfully, they can help foster lasting collaborations, create life-long friendships, and provide a safe harbor within academia. Unfortunately, they can also be stress-inducing and counterproductive if not managed with care. Here, we have provided 10 simple “rules” that have worked for our lab group. We acknowledge that the needs of lab groups differ and that lab missions can change over time. It is important to revisit objectives, rules, and roles (be flexible and adaptable!) as group membership changes. Adopting the 10 simple rules suggested above will go a long way to making your lab meetings more productive and enjoyable. Ultimately, our lab meetings, conducted with meeting guidelines, have created a refuge in the face of chaos and isolation, providing community and belonging while maintaining professional productivity. Although Rules 1 to 9 lay out the importance of having rules and guidelines, Rule 10 may be the one “to rule them all.”
